# A Review on Automatic Facial Expression Recognition Systems Assisted by Multimodal Sensor Data

**DOI:** 10.3390/s19081863

**Published:** 2019-04-18

**Authors:** Najmeh Samadiani, Guangyan Huang, Borui Cai, Wei Luo, Chi-Hung Chi, Yong Xiang, Jing He

**Affiliations:** 1 School of Information Technology, Deakin University, Burwood, VIC 3125, Australia; guangyan.huang@deakin.edu.au (G.H.); bcai@deakin.edu.au (B.C.); wei.luo@deakin.edu.au (W.L.); yong.xiang@deakin.edu.au (Y.X.); 2 Data61, CSIRO, Battery Point, TAS 7004, Australia; chihung.chi@data61.csiro.au; 3 Swinburne Data Science Research Institute, Swinburne University of Technology, Hawthorn, VIC 3122, Australia; jinghe@swin.edu.au

**Keywords:** facial expression recognition (FER), multimodal sensor data, emotional expression recognition, spontaneous expression, real-world conditions

## Abstract

Facial Expression Recognition (FER) can be widely applied to various research areas, such as mental diseases diagnosis and human social/physiological interaction detection. With the emerging advanced technologies in hardware and sensors, FER systems have been developed to support real-world application scenes, instead of laboratory environments. Although the laboratory-controlled FER systems achieve very high accuracy, around 97%, the technical transferring from the laboratory to real-world applications faces a great barrier of very low accuracy, approximately 50%. In this survey, we comprehensively discuss three significant challenges in the unconstrained real-world environments, such as illumination variation, head pose, and subject-dependence, which may not be resolved by only analysing images/videos in the FER system. We focus on those sensors that may provide extra information and help the FER systems to detect emotion in both static images and video sequences. We introduce three categories of sensors that may help improve the accuracy and reliability of an expression recognition system by tackling the challenges mentioned above in pure image/video processing. The first group is detailed-face sensors, which detect a small dynamic change of a face component, such as eye-trackers, which may help differentiate the background noise and the feature of faces. The second is non-visual sensors, such as audio, depth, and EEG sensors, which provide extra information in addition to visual dimension and improve the recognition reliability for example in illumination variation and position shift situation. The last is target-focused sensors, such as infrared thermal sensors, which can facilitate the FER systems to filter useless visual contents and may help resist illumination variation. Also, we discuss the methods of fusing different inputs obtained from multimodal sensors in an emotion system. We comparatively review the most prominent multimodal emotional expression recognition approaches and point out their advantages and limitations. We briefly introduce the benchmark data sets related to FER systems for each category of sensors and extend our survey to the open challenges and issues. Meanwhile, we design a framework of an expression recognition system, which uses multimodal sensor data (provided by the three categories of sensors) to provide complete information about emotions to assist the pure face image/video analysis. We theoretically analyse the feasibility and achievability of our new expression recognition system, especially for the use in the wild environment, and point out the future directions to design an efficient, emotional expression recognition system.

## 1. Introduction

Today, the majority of our time is spent on interacting with computers and mobile phones in our daily life due to technology progression and ubiquitously spreading these mediums. However, they play an essential role in our lives, and the vast number of existing software interfaces are non-verbal, primitive and terse. Adding emotional expression recognition to expect the users’ feelings and emotional state can drastically improve human–computer interaction (HCI) [1,2]. HCI has been considered as one of the most attractive and fastest growing fields, and consequently, human–robotic interactions (HRI) and healthcare systems have been remarkably and rapidly developed [3,4].

Humans usually employ different cues to express their emotions, such as facial expressions, hand gestures and voice. Facial expressions represent up to 55% of human communications while other ways such as oral language are allocated a mere 7% of emotion expression [5]. Therefore, considering facial expressions in an HRI system enables simulation of natural interactions successfully. Indeed, robots can easily interact with humans in as a friendly a way as possible when they can analyse facial expressions and figure out their emotional states. In this way, they can be used in a healthcare system to detect humans’ mental states through emotion analysis and improve the quality of life. The mental states are unfolded in daily situations where robots can inspect positive and negative emotions. Positive facial expressions, such as happiness and pleasure, demonstrate healthy emotion states while unhealthy emotion states are represented by fetching negative facial expressions (e.g., sadness and anger). An efficient facial expression system (FER) can significantly help people to improve their mental emotion state by exploring their behaviour patterns. McClure et al. [6] and Coleman et al. [7] have shown that some mental diseases such as anxiety or autism are diagnosed by investigating the emotional conflicts, which appear on the patients’ expressions.

An efficient FER plays a crucial role in computer graphics where human faces had to been modelled and parameterized as avatars and computer animations [8] by accurately characterizing of face geometry and muscle motion. Many technologies such as virtual reality (VR) [9] and augmented reality (AR) [10] employ a robust FER to implement a natural, friendly communication with humans.

The majority of FER systems attempt to recognize six basic emotional expressions including fear, disgust, anger, surprise, happiness, and sadness introduced by Ekman [11]. A few FER systems define more emotions such as contempt, envy, pain and drowsiness. According to the extensive usage of FER systems in various fields, the researchers have considered them from a variety of views. Some FER systems are grouped into two types: spontaneous and pose-based. Spontaneous FER systems detect the facial expressions explicitly appearing on people’s faces when they deal with daily situations, such as during dialogues and while watching movies. On the other hand, pose-based expressions refer to artificial expressions which people mimic when they are asked to do in a sequence [12]. It is demonstrated that the appearance and timing of posed expressions may help differentiate them from spontaneous expressions [13]. Other FER systems are categorized into micro-expression and macro-expression detectors. Micro-expressions indicate hidden expressions, which are quick (lasting less than ½ second), brief and difficult to understand by humans, and individuals try to hide their pure emotions, especially in high-stake situations [14]. Ekman has asserted that micro-expressions may be considered as the best cues for lie detection [15]. In contrast, macro-expressions last ½ second to 4 seconds and occur in our daily interactions with others.

There is a list of sensors such as camera, eye-tracker, electrocardiogram (ECG), electromyography (EMG), and electroencephalograph (EEG) which are employed in different FER systems. However, the camera is the most popular sensor due to its availability and simple usage. In this paper, our survey focuses on different multimodal sensors used in FER systems, and we explain how their different inputs can be combined to improve the performance of FER systems.

**Figure 1:**
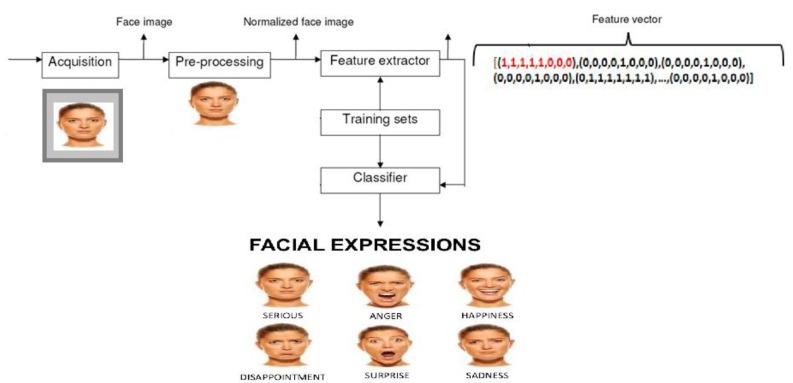
The different stages of facial expression recognition (FER) system.

An ordinary camera-based FER system consists of three main stages: pre-processing, feature extraction, and detection (as shown in Figure 1). At the first stage, the face region is recognized from an input image, and the landmarks such as eyes and nose are discerned from the face area. Then, the necessary features are extracted from the face to facilitate a recognition phase, and various expressions are finally classified at the third phase [16].

Although many techniques have been developed for FER systems, there are no comprehensive surveys on the FER systems that use multimodal sensors. Although Corneanu et al. have reviewed 3D, thermal, and depth video approaches [17], they have not concentrated on real-world approaches using multimodal data and other existing data formats such as EEG signals. Some reviews [18,19,20] have focused on traditional methods while a few [21] have analysed new approaches such as deep learning. Recently, Mehta et al. have proposed a survey of FER in mixed reality [22]. However, the abovementioned surveys of the FER systems present methods and technologies that only process data caught from one sensor (mostly camera). The authors have considered another perspective such as automatic analysis of face and surveyed the important cues for interpreting facial expressions [23]. Many surveys focus on video-based FER systems [24,25] and 3D facial expression recognition [26] while only a limited number focus on the techniques used in real-world FER [27]. Therefore, this paper mainly introduces the existing approaches that tackle the challenges faced by FER systems for real-world use; that is, how different multimodal sensors are explored to improve the performance of FER systems especially for the extreme and wild environment. Our study also covers the techniques employed in multimodal FER systems and benchmark datasets. The contributions of this survey are listed below.

- We introduce a variety of sensors that may provide extra information and help the FER systems to detect emotions and focus on studying using multimodal sensors to improve the accuracy and reliability of an expression recognition system.
- We comprehensively discuss three significant challenges in the unconstrained real-world environments, such as illumination variation, head pose, and subject-dependence and explore the role of multimodal sensors combining with FER systems to tackle the challenges. We comparatively review the most prominent multimodal emotional expression recognition approaches as well as discussing the advantages and limitations of used sensors. In this way, it can be useful for other researchers to find a road map in this area.
- We briefly introduce the public datasets related to FER systems for each category of sensors.
- We design a reliable and robust expression recognition system by integrating the three categories of sensors to image/video analysis, especially for use in the extreme wild environment, and point out the future directions of FER systems.

The organization of the paper is as follows: Section 2 reviews the existing FER systems and their advantages and disadvantages. We have explored them according to their evaluation in lab-controlled or real-world environments and have categorized the related challenges into three main groups. Section 3 explains three multimodal sensors employed in FER systems. The existing approaches related to each categorization are reviewed and their limitation, pros and cons, are discussed. In Section 4, the benchmark data sets are described and grouped according to the relevant multimodal sensors. In Section 5, the described approaches are discussed and compared together. The feasibility of the proposed method is theoretically analysed to solve the mentioned challenges in existing FER systems for real-world use by combining various data from different sensors in Section 6. Also, future directions and open problems are discussed. Finally, this paper is concluded in Section 7.

## 2. Motivation

FER systems have broad applications in various areas, such as computer interactions, health-care systems and social marketing. However, facial expression analysis is incredibly challenging due to subtle and transient movements of the foreground people and complex, noisy environment of the background in the real-world images/videos [28]. There are three main challenges caused by illumination variation, subject-dependence, and head pose-changing; these widely affect the performance of the FER system. The state-of-the-art techniques in FER systems are effective for use in controlled laboratory environments but not for applications in real-world situations.

Extracting features is a key stage in an FER system for detecting emotions. An FER system robustly performs when the extracted features can lessen the within-class variations and maximise between-class variations. Good feature representation can guarantee an efficient and accurate recognition process. There are two types of feature-based facial expression recognition techniques [29,30,31,32,33,34]; geometric-based and appearance-based. The former refers to the FER systems, which extract local facial features including shape, positions, and angles between various facial elements, i.e., ear, eye, mouth and nose, and the feature vector is illustrated based on the geometrical relationship. The latter refers to the FER systems, which describe the appearance and employ texture information of face as a feature vector. The appearance-based approaches can obtain higher recognition rate and are more popular than geometric-based methods since it is a complicated task to find the efficient and proper geometric features in unconstrained environments and real-world applications. Wang et al. have comprehensively reviewed the facial feature extraction methods and pointed out the challenges brought by unconstrained or real-world environments [35].

Classification of the emotions is the second key stage in an FER system. The feature vectors are employed to train the classifier, which is used to assign one of the expression labels to the input face. High-dimensional feature vector challenges the face analysis, which impacts the performance and speed of FER systems. Therefore, various feature reduction techniques, such as PCA [36], are used to reduce the dimension of features for improving computation efficiency, especially for designing a real-time FER system. While some camera-based FER systems have focused on the static and single face image, other approaches can handle videos in real-world and dynamic scenarios. A video-based FER system can be categorized into frame-based and sequence-based. A frame-based FER system applies a single frame to detect different facial emotions, and frame sequences are utilised to extract temporal information in the different frames and recognize facial expressions. Many classification methods have been employed in the FER systems, such as support vector machine (SVM) [37], random forest [38], AdaBoost [39,40], decision tree [41], naïve Bayes [42], multilayer neural networks and K-nearest neighbours [43], hidden Markov model (HMM) [44] and deep neural networks [45]. Although researchers have attempted to solve the challenges in the real-world applications using existing classification techniques to train classifiers, there is not a comprehensive, reliable and robust classification method for the FER systems in real-world conditions that can handle three main challenges (illumination variation, subject-dependence, and head pose).

### 2.1. Illumination Variation

One of the common issues in FER systems is the variation in illumination levels which may affect the accuracy of extracting face features. Researchers have attempted to improve robustness in the shifting lighting conditions in various stages of the FER systems.

Liu et al. have proposed an FER system which could extract features in a wild environment [46] using several kernels on Riemannian manifold and three different classifiers (kernel SVM, logistic regression, and partial least squares). Two features (HOG and dense SIFT) were developed to enable the system to be resistant to illumination variation. Also, the authors have employed nine-layer models of a deep convolutional neural network to extract the third group of features. By evaluating the system with an AFEW dataset, the accuracy of 50.4% was obtained when the input data was both audio and video. The authors have presented another work to detect seven facial expressions [47] and modelled each expression as a spatiotemporal manifold (STM) by dense low-level features. Then, a universal manifold model (UMM) was employed to describe all STMs. A multi-class linear SVM was used to classify the expressions and the accuracies of 94.19% (CK+ dataset), 75.12% (MMI dataset), and 31.73% (AFEW dataset) were obtained. The proposed method is not suitable for real-time applications due to implementing a deep network when the resources are limited, for instance, such as being used in mobile phones.

Principal component analysis (PCA) and ICA have been widely employed to demonstrate facial expressions [48,49,50], but they are not resistant to illumination variation. Hence, local binary patterns (LBP) [51,52] have been developed in FER systems due to less computational time and better tolerance than ICA when the illumination varies in the environment. Local directional pattern (LDP) [39], which represents the gradient features or facial edges, is demonstrated robust against illumination variation. A real-time FER system has been proposed by Ding et al., which is resistant to changing lighting [53]. They have used double LBP to reduce dimensions, applied logarithm-Laplace (LL) domain to achieve robustness and utilized Taylor feature pattern (FTP) based on LBP and Taylor expansion to extract the optimal facial feature from the Taylor feature map; these techniques helped to achieve an accuracy of 92.86% (CK+ dataset). While they could drastically reduce the feature dimension to only 24-dimensional size, feature extraction time of Alexnet (20.213 ms) is much less than the proposed method (54.43 ms). However, it outperforms Alexnet in recognition rate and space complexity. Uddin et al. have presented a modified local directional pattern (MLDP) to improve LDP by considering directional signs per pixel [54], applied generalized discriminant analysis (GDA) to overcome lighting shift and added depth information to the video; this method could increase the accuracy from 93.3% to 96.25%. Also, they have used Deep belief network (DBN) as a classifier to perform faster than a common deep neural network (DNN). However, it is not suitable for running in a general system without a GPU. Gupta et al. have proposed a new adaptive filter using deep neural networks [55] to detect the facial expressions, which reduced the high dimensional videos data by 4D convolutional layers, used a semi-supervised training manner to assign labels to unlabelled data and added a new normalization layer to control lighting varying within the network; this system achieves the accuracies of 65.57% (MMI dataset), 90.52% (CK+ dataset), and 51.35% (Florentine dataset) under standard conditions and the accuracies of 59.03%, 73.68%, and 48% under changing lighting. Although the method can handle the illumination variation, it suffers from limitations of head pose and constant input video frames. Applying higher resolution videos (more frames) increases time computation.

Fast Fourier Transform and Contrast Limited Adaptive Histogram Equalization (FFT+CLAHE) has been used to overcome poor lighting conditions [41]. The authors have computed merged binary pattern (MBPC), merging of local features per pixel, applied PCA to reduce the feature dimensions and implemented several classifiers, such as KNN, decision tree, simple logistic, SMO and MLP, to classify different facial expressions. This method achieves the best accuracy of 96.5% for the SFEW dataset. PCA could reduce 4608 extracted features to the minimum number of 60 which made the proposed FER system more proper for real-time applications. Also, reasonable accuracy rate using simple, quick classifiers demonstrates it as an excellent real-time application. Table 1 summarizes the state-of-the-art FER systems which tried to tackle this challenge.

### 2.2. Subject-Dependence

Subject-dependence means that an FER system cannot generally detect the facial expressions and is only able to recognize the expressions of pre-trained human faces. Handling this challenge requires a reliable classifier or huge datasets including many faces with various natural dissimilarities and many efforts have been done to present individual-independent FER systems. Researchers should develop the algorithms that model the face features and patterns instead of employing some methods such as PCA, and LBP since these methods missed the key features. A spatial–temporal network-based FER system has been proposed by Zhang et al. [56] to tackle this issue. To extract temporal, geometric features of the face, a part-based hierarchical recurrent neural network (PHRNN) was used to model the facial morphological variations, a multi-signal convolutional neural network (MSCNN) was employed to find the spatial features of face, and loss functions were used to maximize the variations of facial expressions; this method achieves the accuracies of 98.5% (CK+ dataset) and 81.18% (MMI dataset). The authors have tested the proposed model on a standard platform included Intel^®^ Core™ i7-4770K CPU @ 3.5 GHz and GeForce GTX TITAN GPU. The feature extraction step has executed 909 FPS (Frame per Second), and the classifier has run 367 sequences per second. In this way, it is a suitable FER system for real-time applications. Uddin et al. have employed depth video to overcome person independent expression recognition [57]; the method is detailed in Section 3.2.3. Table 2 summarizes the existing methods for solving the subject-dependence challenge.

### 2.3. Head Pose

As mentioned before, most existing approaches focus on lab-controlled conditions in which the faces are often in the frontal view; but in real-world environments, the frontal view is not always available and, thus, causes a challenge in detecting the facial expressions. Only a few approaches have considered different aspects of views but with very low accuracy. In [58], geometric features were extracted from the warp transformation of facial landmarks to detect facial shape variation, and dynamic facial textures were extracted by Histogram of Oriented Gradients from Three Orthogonal Planes (HOG-TOP) features to enable the system to track facial movements; this method achieves the accuracy of 46.8% (AFEW dataset). The proposed approach executed in 27 ms on 64-bit Win 7 operating system and a Core i7 CPU. Therefore, it is possible to use the method in real-time applications without GPU. In [59], two visual features and an audio feature vector are used to design an FER system resistant to face movements and face variation; the method is detailed in Section 3.2.1. Liu et al. have attempted to detect spontaneous smile in the wild environment when the faces are not in the frontal views [60]; the relation is extracted between image patches and smile strength characterized conditional to head pose by random regression forests, and several regression trees have been combined together to improve the accuracy by training a small dataset augmented using a multi-label strategy. To improve the speed, an ensemble algorithm was developed by selecting only a fraction of trees and achieved the accuracies of 94.05% (LFW dataset) and 92.17% (CCNU-Class dataset). The labelled Faces in the Wild (LFW) dataset [61] that contains 13,232 real-world images with two expressions (neutral and smile) and different head poses are useful in face recognition, and the CCNU-Class dataset consists of 500 images recorded in real classes. Combining different features extracted from various sensors may help overcome the head pose problem. The execution time of the approach was 9 ms on a computer with Core i7 4.2 GHz CPU which demonstrates its high performance in real-time applications. Table 3 summarizes the state-of-the-art FER systems which tried to tackle this challenge.

## 3. Multimodal Sensors

As mentioned above, the camera is the most popular sensor used in FER systems to capture images and record videos of scenes or individuals. However, when we use a camera-based FER system in the extreme wild real-world environment, three main challenges including illumination variation, head pose, and subject-dependence may impact the performance of the FER system. One solution is to add other dimensions to the camera feature vector, which can be captured from other sensors. By reviewing various approaches, we categorize the sensors used in FER systems to three classes: detailed-face, non-visual, and target-focused sensors. We explain these sensors and related existing approaches to improve the FER system.

### 3.1. Detailed-Face Sensors

In addition to analysing the whole face to detect the emotions, it is useful to find new patterns captured from each section of the face. The human eye is an integral part of the face, which exposes the details of mental statues of individuals, such as presence, attention, focus, drowsiness, and consciousness. It is possible to capture these properties of eyes using an eye-tracking sensor technology that provides information about exactly where the eyes are focused. It assists to better intercommunicate with computers and other devices when the hands’ motions are not recorded as the input and a sign of expressing the emotions.

Combining eye-tracking with other input modalities, for example, keyboard, touchpad and voice, is one of the best methods in different applications such as psychological tests, medical settings and marketing to perceive how attention is distributed. Besides, eye-tracking can assist the FER system to perform better and accurately recognize the facial expressions.

There are a handful of works which have focused on the combination of the eye tracker and other sensors’ input to recognize facial expressions. Emotracker is an application which tries to detect the emotional statues of individuals [62], which comprises two types ofsoftware (Tobii Studio [63] and Noldus Face Reader [64]). These tools keep robustness and accuracy even in real-world environments. For example, 14 users who watched three approximately 2-minute- and-a-half videos were asked to approximate their expressions (amused, scared, disgusted, surprised, angry, sad and other), and a video of user’s face is recorded as well as user’s gaze log and user’s navigation information. Face Reader analyses the recorded video and Emotracker processes the result in addition to the other two user’s information. It produces two maps, “emotional heat” and “emotional saccade”; the former refers to “emotional layers” and the latter indicates the gaze path when the user has been looked at for a minimum time. By processing these maps, the individuals’ emotions are detected, and an emoji (A small digital image or icon to show the emotion) related to the perceived emotion is displayed on the application.

### 3.2. Non-Visual Sensors

#### 3.2.1. Audio

As we know, the camera records audio in addition to video, and it has been proven that facial expressions are correlated well with the body and voice [65,66]. Although previous video-based FER systems only employed visual data to recognize the facial expressions, researchers have recently presented the effectiveness of audio signals in designing an accurate and reliable FER system. However, it is a challenge to find the proper fusion method for combining audio and visual information. Silva and Ng have proposed an FER system, which uses audio and video information to recognize six basic expressions [67]. They have applied a rule-based system to combine the data and created a simple dataset including 144 images and audio files from two individuals. The KNN and HMM were used to classify the emotions, and the average accuracy of 72% was obtained. The number of subjects in the used dataset was small, and larger datasets should evaluate the method. It was claimed that it would perform less than 1 second in real-time applications. Liu et al. have presented an FER system in EmotiW challenge 2014, which employs both audio and video information [46]. They have developed the one-score linear fusion method to automatically select the optimal feature set of audio and video. By applying the method to an AFEW dataset and classifying seven basic emotions by three different classifiers, an accuracy of 50.4% was obtained. The FER system introduced by Yan [59] is one of the recent systems that have employed both visual and audio information. They have proposed a novel collaborative discriminative multi-metric learning (CD-MML) to recognize facial expressions in videos. For the visual feature, there were two types of features: 3D-HOG and geometric warp feature. By extending the traditional 2D HOG [68] and obtaining three orthogonal planes, a HOG feature was extracted from each block located on each plane. Then, these HOG features were combined to form a descriptor for each frame and a high dimensional feature vector finally described whole face video with 1728 dimension. To form a 654-dimensional geometric warp feature vector, the authors have considered the facial motions among adjacent frames and found 109 pairs of triangles with the corresponding vertexes located at 68 facial landmarks. For the audio, 21 functions of acoustic features are applied, and a 1582-dimensional feature vector was formed per each video. PCA was employed to reduce the dimension of each feature vector to 150 dimensions in order to fuse them. This method achieves the accuracies of 46.8% (AFEW dataset) and 96.6% (CK+ dataset). CD-MML was robust to tackle facial appearance variations and over-fitting problems, but it could not perform reliably in real-world environments and it is necessary to find more efficient feature learning methods to improve performance. In [58], an FER system is provided by fusing both audio and visual features, in which a new feature descriptor explained in Section 2.3 to overcome facial motions. The multiple feature fusion technology based on multiple kernel SVM was applied to classify the expressions assisting both audio and video data. The method achieves accuracies of 95.7% (CK+ dataset) and 40.2% (AFEW 4.0 dataset). A brief explanation of each state-of-the-art FER systems which employed non-visual sensors is shown in Table 4.

#### 3.2.2. ECG and EEG Sensors

Four physiological signals, i.e., electrocardiogram (ECG), electromyography (EMG), electroencephalograph (EEG), and Electrooculography (EOG), are commonly used in medical areas. ECG and EEG are the acquisition of electrical activity of the heart and brain, respectively, captured over time by electrodes attached to the skin. By attaching the electrodes to the skin or inserting into them into muscles, the EMG signal is recorded to assess the electrical activity of the muscle [69]. EOG measures the cornea-retinal standing potential that exists between the front and back of the human eye, and it is used to diagnose ophthalmological and to record eye movements [70].

Only a limited number of recent studies are related to ECG-based emotional expression recognition [71,72] while many studies have focused on detection of emotional expressions using EEG signals due to robust sensing of emotions in the brain [73,74,75]. However, high-dimensionality of EEG signals causes a problem so that it is hard to identify the most effective features for emotional expression recognition. So, the average accuracy of EEG-based emotional expression recognition is approximately 72%. Some techniques have been proposed to detect emotions by fusing several physiological signals. Hassan et al. have provided an emotional expression recognition system which fuses physiological signals such as EEG, and zEMG for accurate and reliable recognition of different emotions [76]. Since both user’s non-expressive behavior [77,78] and environmental effect like illumination variation challenge the emotional expression recognition from facial expressions, it seems that combining both facial expressions and physiological signals can improve the reliability and accuracy of FER systems in real-world situations. 

In [79], a system is provided to recognize gender and expressions, which has employed an Emotiv EEG wireless headset to record EEG signals and EyeTribe to track eyes. The poor signal quality, commercial, and off-the-shelf mediums were used rather than high quality, expensive devices in order to propose a low-cost system. Moreover, it can be employed in real-time applications due to running for less than one second. The event-related potential (ERP) and fixation distribution patterns were extracted to describe the optimal features, and various classification methods, such as Naive-Bayes (NB), linear SVM (LSVM) and radial-basis SVM (RSVM) classifiers, were used to detect binary emotions classes (negative and positive). The W_est_ procedure (The process of automatic labeling) [80] was applied to fuse the EEG and eye-based outputs to form a feature vector. Twenty-four facial emotions were taken from Radboud Faces Database (RaFD) in addition to images of 28 individuals from different nationality and used in 10-fold cross-validation to evaluate the proposed method. The average AUC (Area under the curve) was obtained more than 0.6 and it was shown that women quickly and more accurately expressed negative emotions. Deep learning methods can be employed in the future to solve some limitations in pre-processing like ICA and visual rejection in this system.

#### 3.2.3. Depth Camera

Depth camera is a sensor which can allocate pixel intensities in-depth images according to the camera distance. Depth images can provide more robust features rather than RGB images, and they have been consequently employed in different computer-aided applications, such as body motion recognition [81,82,83] and face detection [84,85]. Moreover, it is significantly more challenging to identify subjects using depth data, and human privacy is consequently protected in the data acquisition phase [57]. As each face component is identified in a depth face images, more researchers use depth face images to recognize the expressions than the researchers using other sensors. Uddin et al. have extracted the dominant features for describing face from depth images and employed deep belief network (DBN) with cloud computing to detect the six basic expressions (sadness, surprise, anger, happy, disgust, and neutral) [54]. Per-pixel in-depth image, eight directional strengths were extracted and sorted by top strengths to form robust modified local directional patterns (MLDP) features. Then, a generalized discriminant analysis (GDA) was applied to find better features. The method was evaluated by a dataset including 40 videos consisted of 10 frames in a two-fold cross-validation manner, and the average accuracy of 96.25% was obtained. They used a depth camera to solve the noise caused by illumination variation and to present a safe privacy per user. However, the method is only tested on the depth videos of ten subjects.

The authors have employed a similar framework by extracting local directional position pattern (LDPP) to recognize the expressions via depth videos [57]. They have built a dataset based on [86] to evaluate the proposed method in the detection of six basic emotions. The accuracy of 89.16% was obtained by applying the introduced data set in a four-fold cross-validation learning. Cai et al. have employed multichannel features extracted from an RGB-D sensor to provide an FER system robust in uncontrolled environments [87]. They have extracted a histogram of oriented gradient (HOG) descriptors based on facial texture features from RGB, depth images, and geometric features of RGB images based on an active appearance model (AAM). The multichannel feature vector has been constructed by combining these descriptors. Besides, they have optimized the parameters of normal SVM using a grid search method and called it GS-SVM. This classifier can perform reasonably under limited resources in real-time applications. Finally, the proposed method has been evaluated by a merged dataset that consists of five expressions (smile, sad, yawn, angry, neutral) captured from EURECOM, IIIT-D Kinect RGB-D, and RGB-D dataset of Aalborg University and the average accuracy of 89.46% was obtained. It was a robust FER system which could overcome the challenges such as head poses and illumination variation. However, it should be tested by other classifiers such as ANN, BN, HMM, and CRF and extended to recognize seven basic expressions.

### 3.3. Target-Focused Sensors

As mentioned above, there are numerous FER systems which employ visual information to recognize the facial expressions. In uncontrolled environments, the illumination changes with time and location, which significantly affect facial appearance and texture. Infrared thermal images, which compute temperatures distributed in various parts of the image, can be used to tackle the lighting issue in designing a facial expression system. Therefore, various FER systems based on infrared thermal images/videos have been studied for more than 15 years [88,89,90,91,92,93,94,95]. However, they only focused on recognizing the posed expressions on a limited dataset; today, researchers are attempting to extend their studies to provide an FER system applicable in real-world situations. As geometric features illustrated precise shapes and distances of the face, finding the facial geometry is tricky due to the dullness of the thermal image [17]. He and Wang have proposed a method to recognize spontaneous facial expressions using both visual and thermal images [96]. They have extracted two forms of features. The first is the active appearance model (AAM) consisted of facial appearance data and geometric, as well as velocity and the rotational speed of head movements. The second is calculating statistical parameters based on differential temperatures between the peak and start infrared images. In total, 1153 features were extracted from thermal and visual images. For combining these features, a feature-level fusion method based on a Genetic Algorithm (GA) has been presented to find the optimal set of synthetic features. The KNN classifier (K = 7) was employed to detect the six basic expressions in an NVIE dataset by a fivefold cross validation manner. An average accuracy of 63% was obtained which showed the effectiveness of the FER system. As GA was used to select the optimal feature set, the space complexity was reduced while the computation time of feature extraction has increased due to applying the slow evolutionary algorithm, GA. However, using KNN classifier makes the method to be as quick as a real-time technique.

In another work, authors have proposed an FER system which uses infrared thermal videos [97]. After some pre-processing and dividing the face area to four sub-regions, they extracted the sequence features from the horizontal and vertical temperature difference sequences per block. The optimal subset of features was formed employing the analysis of variance (ANOVA) method based on F-values to reduce the time complexity of the system. The method was evaluated by a USTC-NVIE dataset by applying the AdaBoost algorithm with KNN weak classifiers. AN average accuracy of 73% could be achieved to recognize spontaneous facial expressions. Wang et al. have presented a method which can characterize the difference between posed and spontaneous expressions [98]. After extracting 19 geometric features to model spatial facial variation, statistical analysis was used to find the optimal set of features (15-dimentional feature vector). Finally, several Bayesian networks (BNs) were employed, and the accuracies of 74.79% and 85.51% were obtained using SPOS and UTSC-NVIE datasets, respectively. The limited number of features and employing a Bayesian network as classifier show that the proposed approach can be used in real-time applications. Table 5 summarizes the existing FER systems which have employed target-focused sensors.

## 4. Datasets

**Figure 2:**
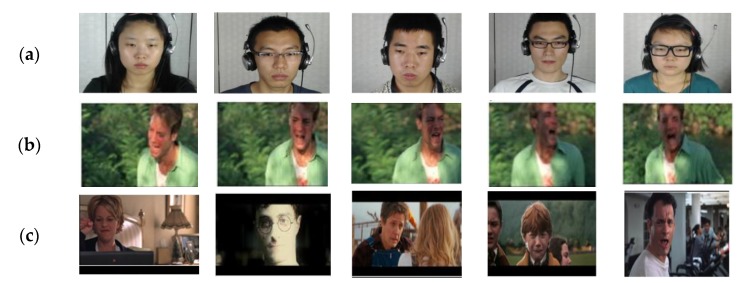
Example datasets: (**a**) CAS(ME)^2^, (**b**) AFEW, and (**c**) SEFW.

Many datasets have been introduced for face analysis while the number of public datasets in the wild environment are few. Researchers have attempted to provide some facial datasets in the wild useful in motion detection [99,100], face recognition [101], and face localization [102]. In this paper, we have categorized the existing datasets according to sensor types. Meanwhile, we have only focused on the camera-based datasets, which were captured from real-world environments or attempted to simulate some real-world conditions in the laboratory. Table 6 and Table 7 summarize the introduced datasets in this paper and Figure 2 shows some examples of CAS (ME)^2^, AFEW, and SEFW dataset. You can find the samples of various datasets including CK+, CMU Multi-PIE, MMI, AM-FED, CAS-PEAL, VAMGS, UTSC-NVIE, and SPOS in the related links provided in Table 6 and Table 7.

### 4.1. Camera-Based Datasets

#### 4.1.1. Chinese Academy of Sciences Macro-Expressions and Micro-Expressions (CAS (ME)^2^)

This dataset consists of both micro- and macro-expressions captured from long videos which were shown to 22 participants. It comprises two subsets: the first contains 87 long videos of spontaneous micro- and macro-expressions. The second entirely includes 357 expression samples: 300 cropped spontaneous macro-expressions and 57 micro-expressions samples. The Action Units (AU) were marked in the expression samples labelled by emotions. They have classified the labels to four groups: negative, positive, surprise, and others. As mentioned before, positive and negative categories demonstrate positive and negative emotions like happiness and sadness, respectively. “Surprise” indicates an emotion which can be positive or negative and vague emotions, which are challenging to classify to six basic emotions, belong to “others” class. In order to have reliable labels, attendees are asked to review the existing labels and to report themselves their emotions. Using a low-rate frame camera for capturing samples makes this public dataset requires an improvement [103]. Figure 2a shows some micro-expression samples of CAS (ME)^2^.

#### 4.1.2. Acted Facial Expression in Wild (AFEW) 4.0

This dataset consists of facial videos recorded in a variety of movies located in real-world situations. It contains three subsets of facial videos; 578 for training, 383 for validation, and 307 for testing. Every face video is labelled by one of the seven expressions (anger, disgust, fear, happiness, neutral, sadness, and surprise). Although there are the original and aligned face videos in the datasets, a pre-processing method introduced by Chen et al. [58] is applied to distinguish each face from each frame in the videos. The AFEW 4.0 is more diverse in faces than other datasets since the videos are captured in natural and real-world environments [104]. Figure 2b shows the samples of this dataset.

#### 4.1.3. Static Facial Expressions in the Wild (SFEW)

This dataset is constructed by selecting the frames from AFEW dataset. SFEW consists of 700 images which are labelled by one of the seven emotions (angry, disgust, fear, happy, sad, neutral, and surprise). It supplies images which contained a variety of challenges of facial expression recognition such as head poses, and illumination variation, broad age range, various faces existed in the real world [105]. Some examples of this dataset are shown in Figure 2c.

#### 4.1.4. Extended Cohn–Kanade (CK+)

This dataset is an extended version of CK dataset and consists of 593 facial videos captured from 123 persons provided by researchers in the University of Pittsburgh. All videos were recorded in the laboratory in controlled situations. Seven emotional expression labels, including anger, disgust, fear, happiness, neutral, sadness, and surprise, were assigned to only 327 videos. At least ten frames were recorded per video where there is a neutral frame, and by progressing to other expressions, it is extended to record a maximum of 60 frames [106]. However, most data are posed expressions and there are 122 spontaneous smiles from 66 persons. The authors attempt to broadcast a new version of the dataset including synchronized 30 degrees from the frontal video in the future.

#### 4.1.5. CMU Multi-PIE

This dataset is an extension of PIE dataset [107] and consisted of 755,370 images captured from 337 subjects who attended in four recording sessions. However, the acquisition data process has been applied in the lab; 19 various illumination conditions were provided while capturing the images. Also, the images were taken from 15 different viewpoints to provide the diversity of the data. They have recorded six expressions per individual (neutral, smile, surprise, squint, disgust, scream) [108].

#### 4.1.6. Florentine Dataset

This dataset contains 2777 video clips which were recorded from 160 subjects. It consists of 1032 clips of posed expressions and 1745 clips of spontaneous expressions. The application was designed in Python language, and many YouTube videos were shown to the subjects to capture seven basic emotions [55,109]. It was claimed that it would be published. 

#### 4.1.7. Autoencoder Dataset

This dataset has been developed to enable very deep neural networks to be trained. It consists of 6.5 million video clips with 25 frames per video and it includes 162 million face images. The clips were captured from various sources, such as YouTube, FOX, CSPAN, and NBC, and Viola–Jones face detector was used to detect and extract the faces. The face landmarks were localized and video clips included faces with the rotation of more than 30 degrees, and quick movement was removed from the dataset [55]. It was claimed that it is the most extensive facial expression dataset and it will be published.

#### 4.1.8. Web-Based Database (MMI)

This dataset consists of 1520 images and videos of posed expressions recorded from 19 subjects. The primary goal of creating it was developing a web-based direct-manipulation application. There are approximately 600 frontal and 140 dual-view images and 30 profile-view and 750 dual-view video sequences which have between and 520 frames. The individuals were asked to express 79 series of expressions, and the lightening conditions and background were changed only for one-fourth of samples [110].

#### 4.1.9. Affectiva-MIT Facial Expression Dataset (AM-FED)

This dataset consists of 242 webcam videos of spontaneous facial expressions collected online from the viewers engaged on the Internet and was filmed while watching one of three amusing Super Bowl commercials. This database was labelled only one expression (smile) and collected of spontaneous expression samples recorded in natural settings. Approximately 695 frames were recorded per video which shows the high resolution [111] and the videos were captured in unconstrained conditions.

#### 4.1.10. CAS-PEAL

This dataset consists of 99,594 images captured from 1040 subjects some of whom wore glasses and hats. It includes five posed expressions in different head poses and 15 various lighting conditions. Also, they wanted to simulate the background variability as it is in the real world, and have employed five different unicolor (blue, white, black, red, and yellow) blankets to make the background. The individuals were asked to express neutral, smile, fear, surprise, to close their eyes and to open their mouth [112]. Furthermore, the images are captured in different illumination conditions and head poses.

### 4.2. Non-Visual Datasets

#### The Vera Am Mittag German Audio-Visual Emotional Speech Database (VAMGS)

This dataset consists of 12 h of audio-visual recordings of the German TV talk show “Vera am Mittag”. The researchers have selected only the images in which the speaker was precisely in front of the camera and consequently collected 1867 images from 20 speakers. Some evaluators (average 13.9) evaluated the images and labelled them by six basic emotions (happiness, anger, sadness, disgust, fear, and surprise). There are two different audio sub-sets: VAM-Audio I contained 499 audio files of 19 speakers which were very clear without any noise such as background music, and VAM-Audio II contained 519 voices of 28 speakers that might have some disturbances [113].

### 4.3. Target-Focused Datasets

#### 4.3.1. Natural Visible and Infrared Facial EXPRESSIONS (UTSC-NVIE)

This dataset consists of posed expressions of 215 individuals. They attended in three experimental sessions with different illumination conditions and were asked to demonstrate six expressions such as happiness, sadness, surprise, fear, anger, and disgust. Moreover, the authors have collected spontaneous expressions by showing various 3–4 min internet videos to the subjects and recorded the visual and thermal information by the corresponding sensors. After watching, they did a self-report to label their emotions. Also, some attendees have worn glasses [114].

#### 4.3.2. Spontaneous vs. Posed (SPOS)

This dataset consists of 84 posed and 147 spontaneous expressions captured from seven subjects five of whom wore glasses. The experimental sessions were in the lab, and both visual and near-infrared information were recorded from subjects. They were shown 14 film clips to express one of six basic expressions (anger, disgust, fear, happiness, sadness, and surprise) and were asked to display each expression twice, resulting 12 posed expression per person [115].

### 4.4. Multimodal Spontaneous Emotion Database (MMSE)

**Figure 3:**
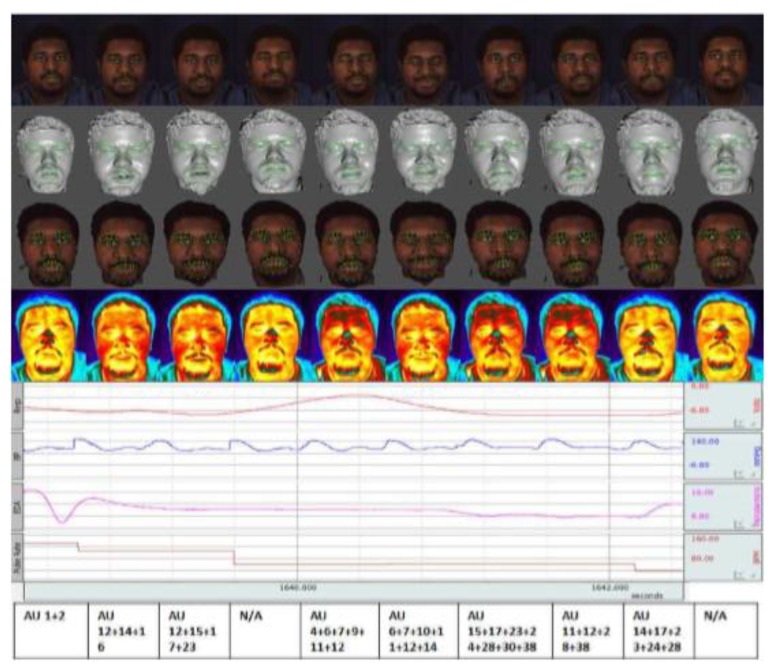
A sample from MMSE dataset which each row from top to bottom shows the 2D image of individual, shaded model, textured model, thermal image, physiological signals, and action units, respectively [116].

This dataset consisted of the data captured from different sensors such as synchronized 3D models, 2D videos, thermal, physiological sequences, facial features, and FACS codes. The heart rate, blood pressure, electrical conductivity of the skin (EDA), and respiration rate were included as a physiological sequence. The data was captured from 140 individuals from various nationalities (east-Asian, middle-east Asian, Hispanic/Latino, and Native American). Ten emotions were recorded per person including surprise, sadness, fear, embarrassment, anger, disgust, happiness, startle, sceptical, and physical pain, and 10 GB data were stored per subject [116]. Figure 3 shows an example of this dataset and different data were collected per person.

## 5. Discussion and Comparison of the Existing Real-World, Multimodal FER Systems

As explained in Section 1, most FER systems focus on recognizing six basic expressions. While the majority of existing systems have achieved high accuracy above 90% in the controlled conditions, real-world applications require more support to improve the accuracy to more than 50% by addressing three main challenges. First, illumination variation, the most common challenge in the wild, has been solved by some researchers. The proposed MBPC [41] could handle pose variation, and slight lighting variation but it is not robust in uncontrollable conditions where various noise levels are added to the images due to illumination variation. However, the authors have tried to manage the illumination variation using STMs [47] and statistical parameters, setting the precise value for many parameters in the real world is a tricky problem which should be tackled. MLDP-GDA is another useful feature extraction method which has been developed to handle the lighting changing [57], but it was only successful in recognizing posed expressions while we need an FER system applicable in real-world environments with many concurrent people who are expressing spontaneous emotions. Taylor feature [53] is another method managing the illumination variation; however, it is resistant to only illumination changing in the laboratory, not in the wild. The recent FER system introduced [55] uses deep learning which requires at least 9 GB of VRAM on the graphics card, and the training process of the system has taken over three days. This computation time and necessary resources do not fit in real-world applications. Therefore, the FER system area needs to support a fast, robust classifier with an appropriate feature extraction method resistant to the unwanted noises to be applicable in the wild.

The second challenge is subject-dependence that there is only real-world research which has focused on it [59]. Using 3D-HOG and CD-MM learning could handle the person-independent and head pose problem in the real world in comparison to single-metric learning methods. However, it needs more efficient features to improve the recognition rate. The FER system [56] aimed to solve the subject-dependence in the lab-controlled environment has the problem of capturing the spatial features when the faces are not in the frontal view. However, MLDP features [57] are more resistant to illumination variation and head pose; using the deep belief network requires much memory resource and high computation.

**Figure 4:**
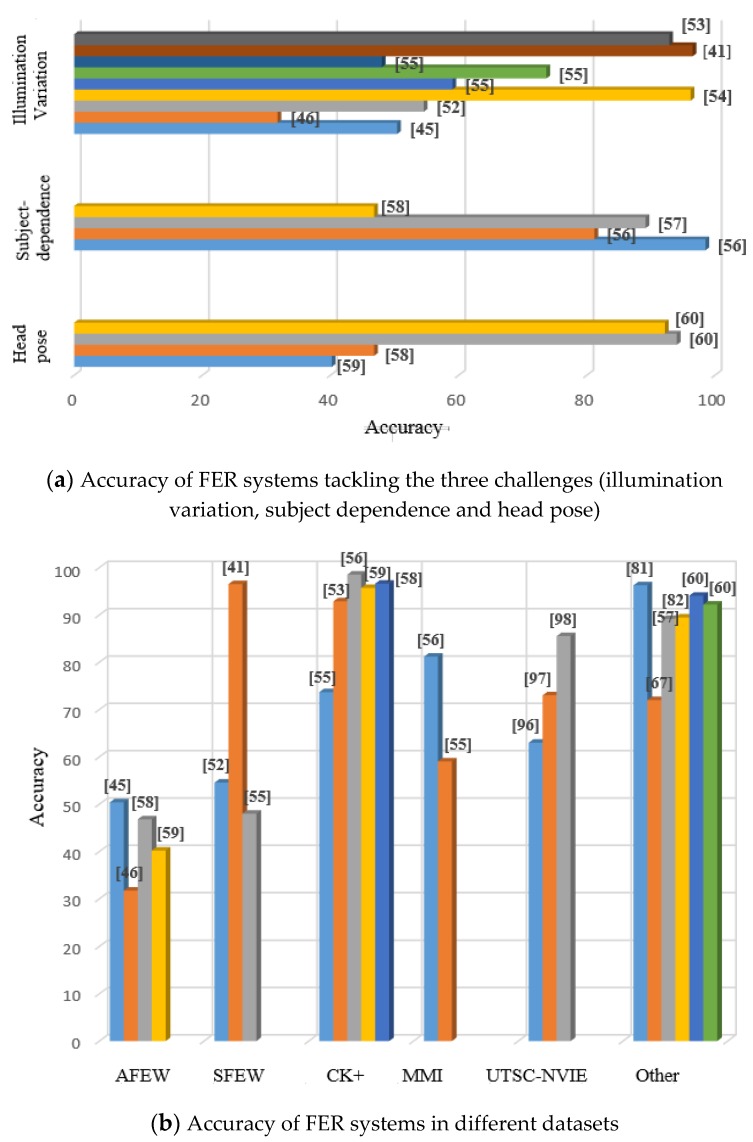
Accuracy of different FER systems.

Head pose is the third challenge which should be addressed for FER systems in the wild. Among the existing methods, it seems that extracting geometric features could make the robust features tackle the pose variation, but researchers should focus on selecting more proper features to enable the FER system to perform in the unconstrained environments. Figure 4a compares the accuracy of different FER systems which have addressed the mentioned challenges. It is noticeable that most studies have considered illumination variation while a few works have focused on two other issues.

The high accuracies in the figure were obtained by the studies which have simulated real-world situations (the mentioned challenges) in the laboratory. In summary, illumination and considerable head pose variation make the FER system challenging in real- world environments, and spontaneous facial expressions usually have low intensity. Moreover, multiple persons may simultaneously express their emotions. Therefore, it is necessary that the researchers concentrate on group facial expression recognition [117,118].

After discussing challenges, we described several types of sensors which can assist the FER systems in recognizing expressions. Although the number of multimodal FER approaches is small, some works have focused on combining different data formats to address the mentioned challenges.

Using audio features along with RGB images is the most common multichannel method in the FER systems. However, the authors have attempted to handle the challenges by adding a feature dimension to RGB features, some other cases such as the fusion method should be considered to design a reliable, robust FER system in the wild. Among existing approaches, PCA has been employed to combine the visual and audio features [119]. As mentioned above, PCA is not resistant to illumination variation, and head pose in real-world conditions. Others have used multi-kernel SVM to fuse audio and HOG-TOP features extracted from the video [57], but it forms only a robust feature set in lab-controlled environments by preserving person-dependent characteristics. Extending the extracted features by another reliable fusion technology can make a robust FER system. Also, fusing infrared-thermal and RGB features have been done by multiple genetic algorithms [96]. Extracted features by another reliable fusion technology can make a robust FER system. Also, fusing infrared-thermal and RGB features has been done by multiple genetic algorithms [96]. It was successful in recognizing the expressions of faces with various views but could not manage subject-dependence. Changing similarity measures used in fusion technology can improve the recognition rate. As a result, adding single dimension (for example audio or depth) to RGB images/videos is not enough for handling all the mentioned challenges and all of them should be combined for having a robust, reliable FER system.

Finally, we explained the benchmark datasets in various sensor categories. As we have concentrated in the real-world applications, the camera-based datasets were introduced which are captured from the wild or in the laboratory by simulating the real conditions such as illumination variation, and head pose. Among them, the AFEW dataset is a comprehensive real-world dataset that includes video and audio from seven emotions. CK+ is the most common dataset captured in the laboratory and provided excellent controlled conditions; the existing methods could achieve the accuracy of more than 98%. A novel dataset called Autoencoder included 6.5 million video clips and has been proposed to demonstrate real-world environments. If it is to be published for the public, it would become the largest real-world dataset. Among target-focused datasets, UTSC-NVIE data were collected in the laboratory by simulating various real-world lighting changing. It is a comprehensive thermal-infrared dataset that includes spontaneous and posed expressions. To perform multimodal sensors in the FER system, there is a multimodal dataset consisting of RGB images, audio, physiological signals, depth, and thermal data. However, it included ten different expressions; the only problem related to it is that the data are captured in the laboratory. Therefore, developing a multimodal dataset in the wild is necessary for the FER area. Figure 4b shows a bar chart which compares the accuracy of the mentioned FER systems according to the used dataset. It is clear that the accuracy of FER systems using real-world datasets is remarkably lower than other systems, even the ones have tried to simulate the real-world conditions. Moreover, many studies have employed the CK+ dataset rather than other datasets. The high performance is seen in the “other” column which illustrates the works applied using non-public datasets.

Another critical factor in FER systems that plays a significant role in real-world conditions is efficiency. Different factors such as computational time and space complexity influence the efficiency of a system, and they can be considered in various steps of the FER system. Some mentioned feature extraction algorithms, for instance, Gabor feature and LBP have computational, and space complexity, respectively, and are not proper to employ in real-time. Using different reduction methods such as PCA, and pyramid LBP [120] can improve the performance of the FER system. Using depth information instead of 3D data captured by traditional 3D sensors improves the computation costs and is more appropriate in real-time applications [121]. Furthermore, recognizing micro-expressions in high accuracy by applying depth information of face is more feasible than applying RGB images. Recently, deep learning methods such as CNN and DBN have attracted many researchers to extract features while they cannot run at a reasonable speed on a general system without GPU. In the classification phase, SVM is the most suitable classifier in real-time conditions among other classifiers. However, its misclassification rate is high when the input data involve the mentioned challenges. The researchers should mostly focus on investigating new algorithms for extracting the appropriate features and try to handle the efficiency in different steps of the FER system.

## 6. Multimodal Sensors Assisting in Automatic Facial Expression Recognition

### 6.1. The Proposed Method and Future Direction

As mentioned above, although Facial expression recognition (FER) has attracted many researchers due to three main reasons, there is a vast gap between real-world applications and lab-controlled approaches related to face expression analysis. We reviewed three significant challenges which significantly affects the performance. Although several methods have been proposed to address these challenges, the expression recognition rate could not grow over 50%. Firstly, it depends on the extracted features. Since individuals’ faces are extremely diverse according to their nationalities, skin texture according to people’s age, and even modes of expression, it is very tricky to find an appropriate expression descriptor applicable to all people. Many researchers have attempted to tackle this issue by extracting different feature sets (geometric-based and appearance-based) from the face and then combining them. However, in this way, the high-dimensionality brings new problems of time-consumption and significant data processing (requiring massive computing and store resources). So, feature reduction methods may be employed to enable the FER system to perform in a satisfactory speed. However, feature reduction may cause that the main facial features are missed, make it individual-dependent, and it is hard to develop a general system for all people especially in the real-world conditions. While deep networks have recently attracted many researchers due to successfully handling the images and videos and reducing the features, they need powerful, expensive computing sources. Therefore, a feasible solution has been developed for lab-controlled datasets, which extracts standard face features from images or video and combines them with the features extracted from other sensors; but this solution which fuses audio and video extracted from videos in the wild environment, could only reach 50% accuracy due to the mentioned challenges.

**Figure 5:**
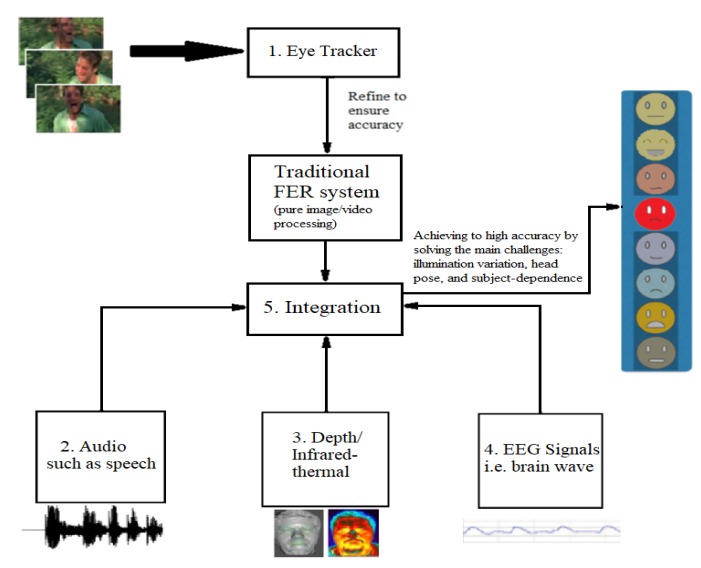
The framework of automatic FER system assisted by multimodal sensor data.

Our vision is to build different feature vectors for different sensors and tackle the challenges one by one using suitable multimodal sensors if need. We designed a framework of an automatic FER system assisted by multimodal sensor data as shown in Figure 5. In this framework, we employ different modules, each addressing one of the existing challenges in real-world wild environments, for improving reliability and efficiency.

(1) Module 1: We use data extracted from detailed-face sensors to improve the performance of the FER system and test the system using real-world background changes, for instance, from indoor to outdoor situations. As an eye tracker can reduce a large amount of face image processing [62], we can use it to efficiently do a coarse detection and thus, image/video processing for detecting and tracking face from other wild background environment is only required to refine the results when confidence is low. This module can improve the efficiency of the proposed method.
(2) Module 2: We collect the audio data along with RGB videos. By adding an audio dimension to the conventional FER system, it is possible to tackle the head pose issue. Works [58,59] have recorded audio and appended several audio features to the feature vector. Accuracy improvement rather than other works in real-world situations demonstrates the usefulness of the new dimension.
(3) Module 3: It is necessary to tackle the most common challenges: illumination variation and subject-dependence. We need to capture depth or infrared-thermal data to add to the usual feature vector. As the works [54,57,87,96,97,98] have focused on extracting depth maps and thermal-infrared information and could achieve to a higher accuracy than other state-of-the-art FER systems in the wild, the results illustrate that appending depth or thermal information to a feature vector can lead the FER system to overcome the mentioned challenges.
(4) Module 4: We record the EEG and ECG data in another module for the situations which the previous modules could not address or could not recognize similar such as disgust and anger. In these positions, using the EEG and ECG signals is helpful due to wave shape variations when people express different emotions. As known, the EEG and ECG signals are unique per individual and can be used as an appropriate feature vector to recognize the emotions [76].
(5) Module 5: We integrate multimodal sensor data with the traditional FER system to classify the expressions with high accuracy.

We will focus on the following three key techniques to implement the above steps. **Multimodal Sensor Data Collection** At the first stage for implementation, we need to collect data including the mentioned multimodal data formats. An MMSE dataset is an excellent data resource, however, the data was only recorded in lab-controlled conditions. For collecting data in real-world situations, we require equipment which can record different types of data and individuals need to easily employ them when they are asking to participate in the experimental sessions. The Eye tracker sensor introduced by [122], the IR-based sensor tracking [123], and EEG signal headsets [124,125] can be used to capture the data in the wild simply. Moreover, the facial expressions would be natural and spontaneous from more subjects to be exactly as the real-world conditions and at least six basic expressions are recorded from each. 

**Fusion Method Selection** After extracting data and providing a dataset, we need to study the cross-correlation of multimodal data and develop a highly accurate fusion technology such as genetic-algorithm based or equal-weighted linear methods to be able to fuse the data in the best manner without missing any essential and key features extracted from different sensors.

**Facial Emotion Classifier** A reliable and accurate FER system requires a robust classification method. By the popular use of deep neural networks and their power in analysing images and videos, they have converted to appropriate, robust classifiers. However, networks with more layers (deeper) perform more accurate and reliable; they are allocated more memory and resources. Therefore, it should be a trade-off between the number of layers and the resources used in real-world applications. Using different classifiers in an ensemble algorithm can handle various challenges, i.e., deep network for managing lighting conditions, and regression random forests for head pose can lead to having a robust detector of a variety of expressions.

### 6.2. Open Problems

**Subject-dependence** Among the mentioned challenges in a facial expression recognition system, subject-dependence is an issue which none of the various sensors can solve. It is very complicated, and an FER system might require a considerable dataset to learn each expression of each particular individual at a specific age. However, the classifier is likely to overfit and, consequently, needs more attention in the future to find the best solution for tackling the problem.

**Large Pose Variations** In the future, by extending the research area and hardware, 3D models can be considered as a proper method to handle large pose variations. Also, acquiring polarization-state imagery of faces in the thermal spectrum can be employed to describe the facial features as it has been shown that it provides more additional textual and geometric information rather than thermal face imagery [126].

**General Multimodal Sensor Data Fusion** Although fusion technology benefits FER systems, finding the appropriate method is more challenging than each issue caused by illumination variation, head pose, and background variability. Many researchers have performed late fusion (i.e., decision-level fusion) while early fusion such as feature-level fusion attracts more support. Therefore, constructing a method to provide the final feature vector formed by different features from various modalities with a different structure, timescales, and metric levels is an open issue in the FER area.

## 7. Conclusions and Future Work

Facial expression recognition (FER) has attracted many researchers in different fields such as human interaction systems, mental disease detection, and affect recognition. However, most applications are applied to controlled laboratory situations, few existing techniques/methods are applicable in the real world, even with meagre recognition rates. We have discussed three main challenges which cause an adverse effect on FER systems: illumination variation, subject-dependence, and head pose. By reviewing state-of-the-art FER systems in the wild environment and explaining the methodologies and limitations, we propose a multimodal sensor framework assisting in facial expression recognition, which theoretically can detect the six basic expressions and improve the accuracy and robustness of the existing systems. We provide a framework of an automatic FER system assisted by multimodal sensor data and theoretically analyse the feasibility and achievability for emotion detection; its effectiveness will probably be demonstrated using real-world experiments in the future. We also point out the open problems in this area that may inspire new approaches to improve the FER systems in the future.

## Figures and Tables

**Table 1:** Existing FER systems for resolving illumination variation.

Classifier	Work	Method	Sensor Type	Dataset	Average Accuracy %
SVM	[46], 2014	HOG, dense SIFT features kernel SVM, logistic regression, partial least squares	RGB Video Audio	AFEW 4.0 (real-world)	50.4
	[47], 2014	STM, UMM features Multi-class linear SVM	RGB Video	AFEW 4.0 (real world)	31.73
Deep Network	[52], 2015	LBP features Convolutional neural network (CNN)	RGB Image	SWEF (real-world)	54.56
	[54], 2017	MLDP-GDA features Deep Belief Network (DBN)	Depth Video	40 videos taken from 10 subjects (Lab-controlled)	96.2
	[55], 2018	Spatio-temporal convolutional neural network	RGB Video	MMI CK+ Florentine (real-world)	59.03 73.68 48
KNN	[41], 2018	FFT-CLAHE, MBPC features KNN, MLP, simple logistic, SMO, J48	RGB Image	SEWF (real-world)	96.5
	[53], 2017	Logarithm-laplace (LL) domain in DLBP Taylor expansion theorem	RGB Video	CK+ (lab-controlled)	92.86

**Table 2:** Existing FER systems for resolving subject-dependence problem.

Classifier	Work	Method	Sensor Type	Dataset	Average Accuracy %
Deep Network	[56], 2017	Deep spatial-temporal networks	RGB Video	CK+ MMI (Lab-controlled)	98.5 81.18
	[57], 2017	MLDP-GDA features Deep Belief Network (DBN)	Depth Video	40 videos taken from 10 subjects (Lab-controlled)	89.16
CD-MM Learning	[59], 2018	3D-HOG, geometric warp, audio features	RGB Video Audio	AFEW 4.0 (real-world)	46.8

**Table 3:** Existing FER systems for resolving head pose problem.

Classifier	Work	Method	Sensor Type	Dataset	Average Accuracy %
SVM	[58], 2018	HOG-TOP and geometric features	RGB Video Audio	AFEW 4.0 (real-world)	40.2
CD-MM	[59], 2018	3D-HOG, geometric warp, audio features	RGB Video Audio	AFEW 4.0 (real-world)	46.8
Regression forest	[60], 2019	Multiple-label dataset augmentation, non-informative patch	RGB Image	LFW CCNU-Class	94.05 92.17

**Table 4:** Existing FER systems using non-visual sensors.

Work	Method	Sensor Type	Fusion Technology	Dataset	Average Accuracy %
[67], 2000	Statistical features, KNN, Hidden Markov Model (HMM)	RGB Video Audio	Rule-based methodology	144 videos from two subjects	72
[46], 2014	HOG, dense SIFT features kernel SVM, logistic regression, partial least squares	Audio RGB Video	Equal-weighted linear fusion technique	AFEW 4.0	50.4
[86], 2016	MLDP-GDA Deep Belief Network (DBN)	Depth Video	No fusion	40 videos included ten frames	96.25
[57], 2017	LDPP, GDA, PCA Deep Belief Network (DBN)	Depth Video	No fusion	40 videos included ten frames	89.16
[79], 2017	ERP, fixation distribution patterns Naive-Bayes (NB), LSVM, RSVM	Eye movements EEG signals	$\mathrm{The}\;W_{est}$ procedure [80]	Radboud Faces Database (RaFD)	AUC > 0.6
[58], 2018	HOG-TOP, geometric features SVM	Audio RGB Video	Multi-kernel SVM	CK+ AFEW	95.7 40.2
[59], 2018	Collaborative discriminative multi-metric learning	RGB Video Audio	PCA	AFEW 4.0 CK+	46.8 96.6
[87], 2018	HOG, AMM GS-SVM	RGB Image Depth Image	Multichannel feature vector	A merged dataset of three public datasets	89.46

**Table 5:** Existing FER systems using target-focused sensors.

Work	Method	Sensor Type	Fusion Technology	Dataset	Average Accuracy %
[96], 2013	Head motion, AAM, thermal statistical parameters KNN	RGB Image Infrared-thermal Image	Multiple genetic algorithms-based fusion method	USTC-NVIE	63
[97], 2013	Sequence features AdaBoost, KNN	RGB Image Infrared thermal video	No fusion	USTC-NVIE	73
[98], 2015	Geometric features Bayesian networks (BN)	Infrared thermal Image	No fusion	USTC-NVIE SPOS	85.51 74.79

**Table 6:** The camera-based datasets (under real-world and simulation of real-world conditions in the lab).

Dataset	Main Feature	Capacity	Emotions	Environment	Link
CAS(ME)^2^	Both spontaneous micro and macro-expressions	87 images of micro and macro-expressions 57 micro-expressions	Four	Lab controlled	http://fu.psych.ac.cn/CASME/cas(me)2-en.php
AFEW 4.0	Videos from real world scenarios	1268 videos	Seven	Real-world	https://cs.anu.edu.au/few/AFEW.html
SFEW	Images from real world scenarios	700 images	Seven	Real-world	https://cs.anu.edu.au/few/AFEW.html
CK+	The most common lab-controlled dataset	593 videos	Seven	Lab controlled	http://www.consortium.ri.cmu.edu/ckagree/
CMU Multi-PIE	Simulation of 19 various lighting conditions	755,370 images	Six	Lab controlled in different illumination variations	http://www.multipie.org/
Florentine	Many participants for collecting videos	2777 video clips	Seven	Lab controlled in different illumination variations	Not still public
Autoencoder	The largest introduced dataset in real world	6.5 million video clips	Seven	Real world	Not still public
MMI	Dual view in an image	1520 videos of Posed expressions	Six	Lab-controlled trying poor illumination conditions	https://mmifacedb.eu/accounts/register/
AM-FED	Webcam videos from online viewers	242 videos spontaneous expressions	Smile	Real-world	http://www.affectiva.com/facialexpression-dataset-am-fed/
CAS-PEAL	Simulation of various backgrounds in the lab	images of posed expressions	Five	Lab-controlled in various illumination conditions	http://www.jdl.ac.cn/peal/home.htm

**Table 7:** The non-visual, target-focused, and multimodal datasets.

Dataset	Main Feature	Capacity	Number of Emotions	Environment	Link
UTSC-NVIE (target-focused)	The biggest thermal-infrared dataset	Videos of posed and spontaneous expressions	Six	Lab controlled	http://nvie.ustc.edu.cn
SPOS (target-focused)	Subjects with different accessories	231 images of spontaneous, and posed expressions	Six	Lab controlled	https://www.oulu.fi/cmvs/node/41317
VAMGS (non-visual)	Visual-audio real world dataset	1867 images of spontaneous expression 1018 emotional voices	Six	Real-world	http://emotion-research.net
MMSE	A multimodal dataset	10 GB data per subject	Ten	Lab controlled	http://www.cs.binghamton.edu/~lijun/Research/3DFE/3DFE_Analysis.html
